# Seed dispersal limitation causes negative legacy effect on restoration of grassland plant diversity on ski slopes

**DOI:** 10.1002/ece3.11654

**Published:** 2024-07-07

**Authors:** Yuki A. Yaida, Taiki Inoue, Tanaka Kenta, Atushi Ushimaru

**Affiliations:** ^1^ Graduate School of Human Development and Environment Kobe University Kobe Japan; ^2^ Sugadaira Montane Research Centre University of Tsukuba Ueda Japan

**Keywords:** afforestation, anemochory, barochory, land‐use change, mowing management, native grassland species

## Abstract

Past forest use often has a long‐term negative impact on the recovery of the original plant composition of semi‐natural grasslands, which is known as a legacy effect. This study investigates the impact of seed dispersal limitations on the restoration of grassland plant diversity on ski slopes with past forest use, highlighting the negative legacy effect on biodiversity recovery. Focusing on ski areas, our research contrasts the vegetation on ski slopes originally created on semi‐natural grasslands such as pasture (pasture slopes) and constructed by clearing secondary forests or conifer plantations (forest slopes). We examined species richness and diversity, considering seed dispersal modes, grassland management history, and seed source proximity. We reveal that the proximity to species‐rich grassland sources is pivotal for the restoration of native grassland vegetation. Particularly, wind‐dispersed species show significant recovery on slopes with sustained management for more than 70 years and those with neighboring species‐rich grasslands, suggesting that both the duration of management and the proximity to seed sources are critical for overcoming the legacy effects of past forest use. Meanwhile, gravity‐dispersed species failed to recover their richness and diversity regardless of both the duration of management and the proximity to seed source grasslands, which their diversity recovered where seed sources neighbored. Our findings emphasize the importance of considering seed dispersal limitation and management history in the restoration and conservation of grasslands and their biodiversity, particularly in landscapes experiencing past human intervention.

## Graphical abstract

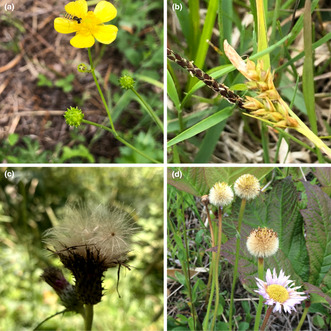

## 1 INTRODUCTION

Changes in land‐use have led to a significant decline and degradation of semi‐natural grasslands around agricultural fields over the past century, attracting global attention (Foley et al., 2005; Newbold et al., 2015; Sala et al., 2000; Török & Dengler, 2018). In particular, land abandonment, and afforestation, which promote forest landscapes, have emerged as primary drivers causing loss of semi‐natural grasslands across the Palaearctic region (Dengler et al., 2014; Török & Dengler, 2018). As semi‐natural grasslands serve as habitats for a diverse range of grassland flora and fauna, their loss and degradation have led to the decline and local extinction of grassland plant species worldwide (Dengler & Tischew, 2018; Karlík & Poschlod, 2009; Petermann & Buzhdygan, 2021). To counter the global decline in the biodiversity of semi‐natural grasslands, conservationists and researchers have begun to preserve these habitats by reintroducing grassland management to abandoned or afforested areas in both Europe and East Asia, thus restoring grasslands (Helm et al., 2019; Koyanagi et al., 2011; Pykälä, 2003).

In regions with a history of different land use, it often takes decades for restored semi‐natural grasslands to regain their original species diversity and composition (Forey & Dutoit, 2012; Helm et al., 2019; Inoue et al., 2021; Yaida et al., 2022). Past human activities, such as afforestation, intensive fertilization and grazing, and land modification (e.g., grading, consolidation, and changes in topography) using heavy machinery, have altered soil characteristics and led to a degraded seed bank, thus having long‐term negative impacts on the recovery of the original plant composition of semi‐natural grasslands (Helm et al., 2019; Matsumura & Takeda, 2010; Roux‐Fouillet et al., 2011; Uchida & Ushimaru, 2014). These long‐term negative impacts, known as “legacy effects,” occur in grassland restoration as well as forest restoration, though the detailed underlying mechanisms are not yet fully understood (Hermy & Verheyen, 2007; Yaida et al., 2022; Yang et al., 2017).

Current land use in ski resort management below the treeline involves maintaining semi‐natural grasslands as ski slopes through annual mowing (Tsuyuzaki, 2002; Yaida et al., 2019). Ski slopes that were originally created on semi‐natural grasslands, such as past pastures (hereafter we refer to them as pasture ski slopes), hold high potential as alternative habitats for numerous native grassland plant species, including endangered plant species (Inoue et al., 2021; Wipf et al., 2005; Yaida et al., 2019, 2022). The grassland species at risk of extinction have been lost over the past few decades in Japan like in countries across Europe (Török & Dengler, 2018; Ushimaru et al., 2018). In contrast, most ski slopes constructed by clearing secondary forests or conifer plantations established on abandoned pastures and mechanically leveling the land (hereafter we refer to them as forest ski slopes) have been known to exhibit lower plant diversity than pasture ski slopes, even a few to several decades after their construction (Inoue et al., 2021; Yaida et al., 2022). This illustrates another example of the “legacy effect” that hinders the recovery of diverse native grassland plants.

The “legacy effect” of forest ski slopes can potentially be explained by two ecological processes: the loss of grassland soil seed banks and the limitations on restoration due to the restricted dispersal of grassland species. Recent studies have shown that even short‐term afforestation can lead to a significant decrease in grassland species richness and vegetation cover (Gallego et al., 2023; Inoue et al., 2021; Prangel et al., 2023). Initially, germination from the soil seed bank can play a crucial role in quickly restoring the original vegetation immediately after forest clearance (Bossuyt & Honnay, 2008; Davies & Waite, 1998; Poschlod et al., 1998). However, the persistence of grassland seed banks over long periods is known to be limited in Europe and East Asia (Bossuyt & Honnay, 2008; Koyama & Uchida, 2022; Koyanagi et al., 2008). When seed banks are lost during forest use, the reestablishment of the original grassland plant communities relies solely on the establishment of new seedlings dispersed from surrounding species‐rich semi‐natural grasslands (Cousins & Lindborg, 2008; Matsumura & Takeda, 2010). Furthermore, the recruitment of seedlings is significantly limited when many species have low dispersal abilities at the seed stage, leading to prolonged restoration periods. Particularly, species with very short dispersal distances, such as gravity‐dispersed species, are expected to be less frequently introduced than those with higher dispersal abilities, such as animal‐dispersed species (c.f. Thomson et al., 2011).

**FIGURE 1:**
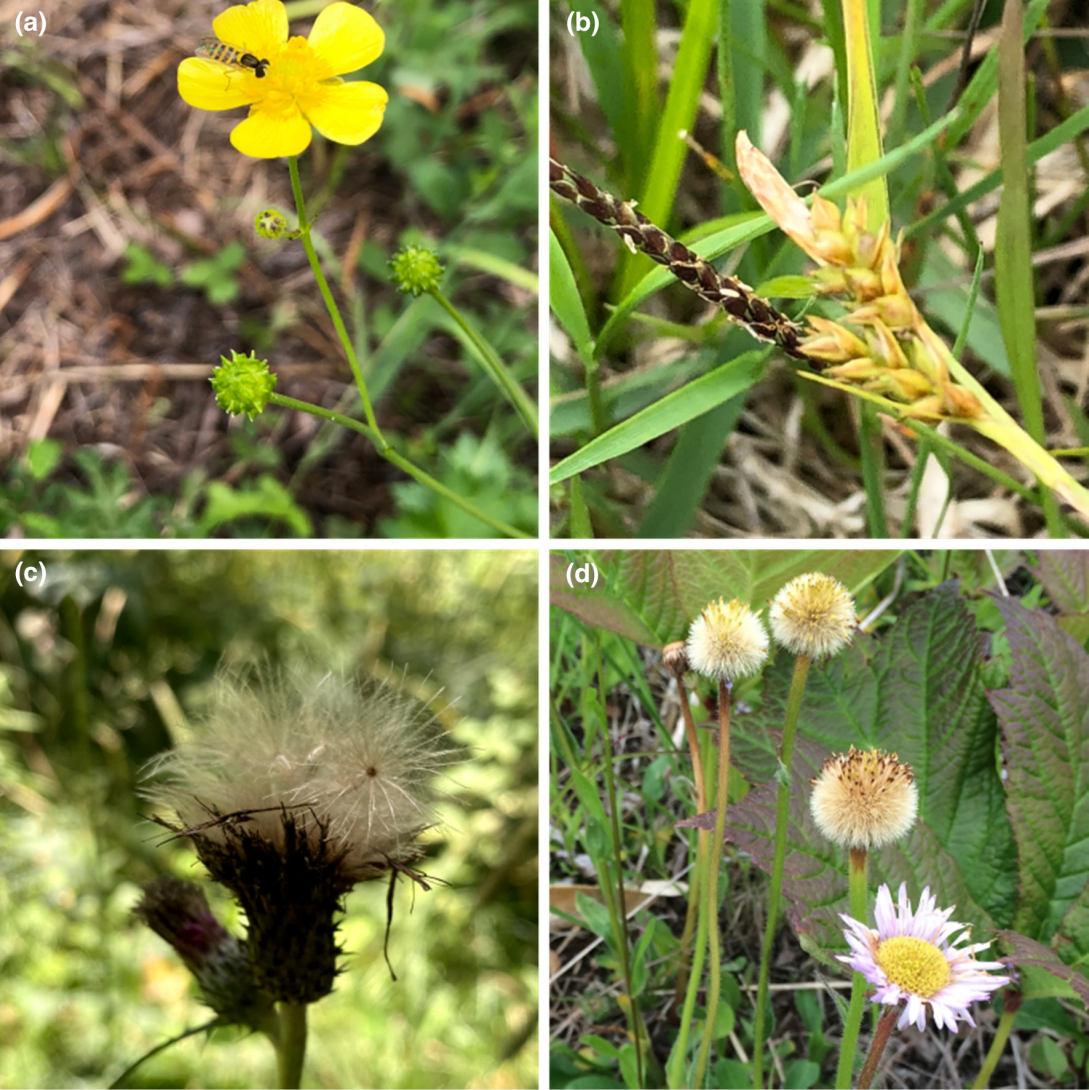
Photographs illustrating the seed traits of grassland plants growing at the study sites. (a) *Ranunculus japonicus* and (b) *Carex nervata* are barochorous (dispersed by gravity), while (c) *Cirsium oligophyllum* and (d) *Erigeron thunbergii* are anemochorous (dispersed by wind).

This study compared the vegetation of forest and pasture ski slopes in the Sugadaira and Minenohara Plateaus, central Japan: Pasture slopes that have been subjected to successive grassland management for at least 100 years and potentially approximately 4000 years (Yamanoi, 1996), and forest slopes constructed by clearing secondary forests and *Larix* plantation in the 1900s and built from the 1940s to the 2000s (Yaida et al., 2022). We surveyed multiple sites with different construction times and spatial positions relative to pasture slopes for forest ski slopes. Previous studies have revealed that even in places that were once pastures, grassland species are hardly distributed in surrounding forests (Inoue et al., 2021), and grassland species do not form a persistent seed bank in the forests in the study area (Yaida et al., 2022). Therefore, it is predicted that forest ski slopes, especially younger slopes or those far from seed source grasslands, will have reduced species diversity due to limited seed supply (the seed dispersal limitation hypothesis). Specifically, it is expected that species that disperse through gravity or their own force, such as gravity‐dispersed (barochorous) and ballistic dispersal (ballochorous) species (Figure 1), will be less frequently reintroduced from distant grasslands to forest ski slopes compared to species with higher seed dispersal abilities, such as wind or animal‐dispersed species. Thus, recovery of short‐dispersed species would be more dependent on area of neighboring seed source grasslands, while that of long‐dispersed species would depend also on time duration since grassland management started. This study aims to elucidate the mechanisms of the “legacy effect” on the restoration process of grassland plants on forest ski slopes by examining the impacts of seed dispersal limitations.

## 2 MATERIALS AND METHODS

### 2.1 Study area

**FIGURE 2:**
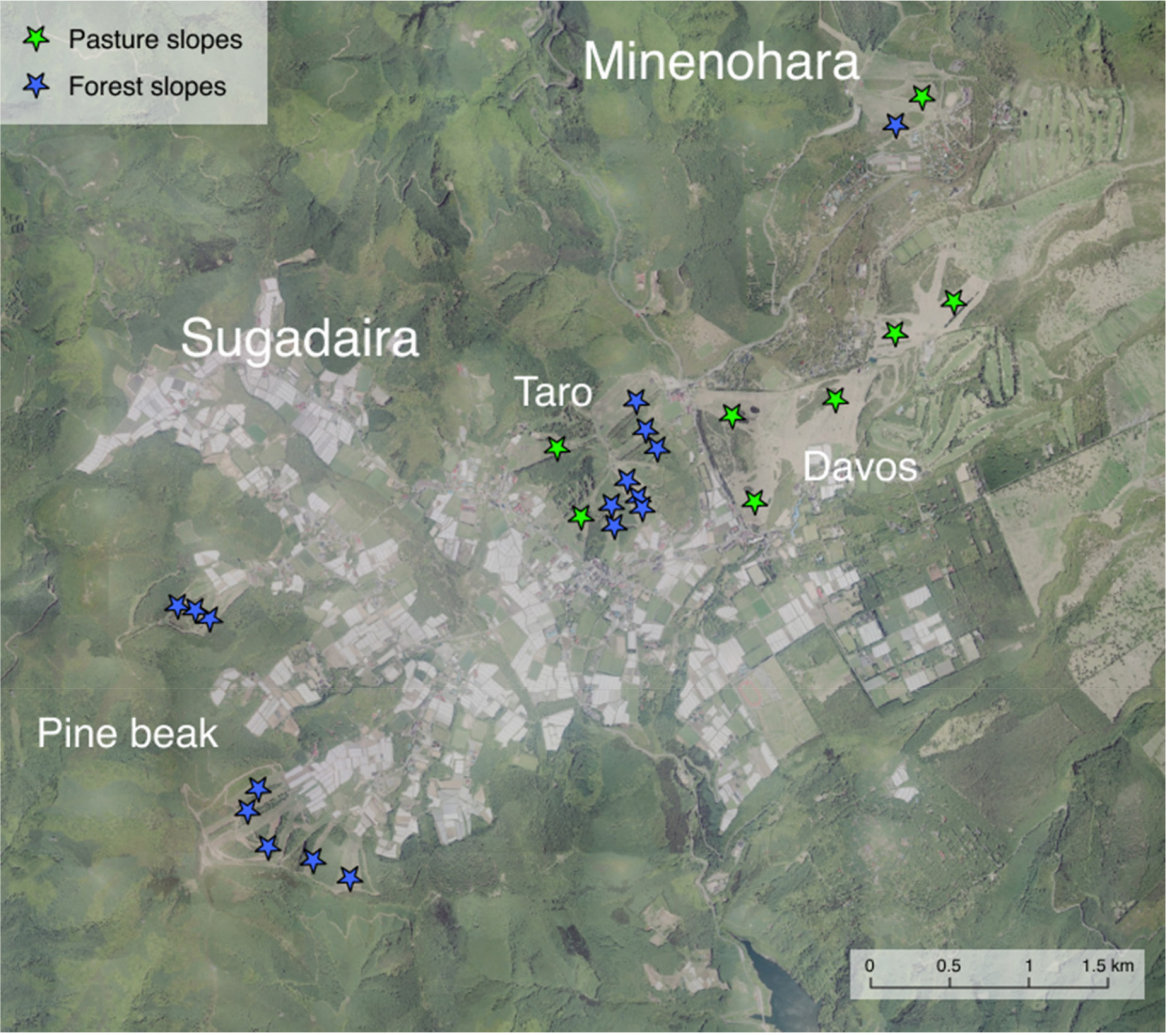
Location of the study area and distribution of study sites: 23 and 2 on Sugadaira and Minenohara Plateaus, respectively. In Sugadaira Plateau, ski slopes are distributed on three areas (Davos, Taro and Pine beak areas). Green and blue stars indicate the sites on pasture slopes and forest slopes, respectively. Each site encompasses four plots positioned at the corner of a 10 m × 10 m square.

This study was conducted at two ski resorts on Sugadaira and Minenohara Plateaus, in Nagano Prefecture, Japan, in 2019 (Figure 2). In the study area, the mean annual temperature was 6.5°C, with a minimum monthly average of −6.2°C in January and a maximum monthly average of 19.4°C in August. The mean annual precipitation was 1219.3 mm during 1989–2018. These meteorological data were recorded by nearby automated meteorological data acquisition systems (36.532° N, 138.325° E, 1253 m above sea level) managed by the Japanese Meteorological Agency.

All study ski slopes are distributed in the cool temperate zone (1327–1882 m a.s.l.), where the climax vegetation consists of broad‐leaved deciduous forests. Semi‐natural grassland conditions have been maintained on all the ski slopes through typically annual mowing in late August or early September in Nagano Prefecture and its surrounding areas (Inoue et al., 2021, 2023; Yaida et al., 2022). Ski slope managers have left cut plant materials in place, allowing litter to accumulate on the slopes (Yaida et al., 2019).

### 2.2 Vegetation survey

The vegetation survey was conducted on 25 ski slopes across the two ski areas (23 and 2 slopes in Sugadaira and Minenohara‐Kogen ski resorts, respectively; Figure 2). On each slope, we established four 1 × 1 m square plots at the corners of a 10 × 10 m square quadrat. In total, we examined 100 plots in the study area. Vegetation surveys were conducted in late June for each plot in 2019. During the survey of each plot, we recorded the abundance of all vascular plant species. To estimate their abundance, each plot was divided into 16 subplots (0.0625 m^2^ squares), and we counted the number of subplots where the species was observed (Ohara & Ushimaru, 2015; Uematsu & Ushimaru, 2013). Given that our study focuses on restoring native grassland plant species, we decided a preferable habitat (grassland or forest) of each species by referring to “Wild Flowers of Japan Herbaceous Plants I–III” and “Woody Plants I–III” (Satake et al., 1981). Grassland species included not only herbaceous species but also shrubs that prefer grassland habitats. For each grassland species, we predicted that the effect of dispersal limitation on reestablishment would vary based on their dispersal modes. We referred to descriptions of seed and fruit traits in the field guides (Satake et al., 1981) and confirmed traits through our field observations. Specifically, we classified species with a mechanism that instantly disintegrates and automatically ejects seeds when the force of the pericarp trying to rupture exceeds the force holding the pericarp together as ballochory. Species with elaiosomes on their seeds were classified as myrmecochory, while those with pappus or wings on their seeds were classified as anemochory. Species that form sap fruits were classified as endozoochory, and those with structures such as thorns and sticky materials on their seeds were classified as epizoochory. Additionally, splash dispersal (dispersed by rain drop) was only confirmed in *Gentiana zollingeri*, classified as hydrochory. All other species were classified as barochory (gravity dispersal). We also summarized data of forest species including forest trees and shrubs and forest floor herbs to compare patterns of species richness and diversity between grassland and forest species.

We measured the maximum height of aboveground plant parts (cm) at the center and four corners of each plot and calculated a mean value per plot as vegetation height for use in the following analyses. Hence, vegetation height is highly correlated with aboveground biomass in Japanese grasslands (Nagata & Ushimaru, 2016; Uchida & Ushimaru, 2014), serving as an indicator of competition among aboveground plants after colonization.

### 2.3 Land‐use history and duration of management as semi‐natural grassland

We examined the land‐use history of each of 100 plots by using cadastral maps drawn from 1910 to 1912 and from 1930 to 1937, as well as aerial photographs from 1948, 1949, 1965, 1975, 1991, 2010, and 2019, provided by the Geospatial Information Authority of Japan. This historical land‐use data has been modified based on Inoue et al. (2021) by adding aerial photographs from 2010. Using this information, we identified that 32 plots had been directly converted from pastures [referred to as pasture (ski) plots] (Figure 2), where grassland conditions have been continuously maintained for at least 100 years and likely for ca. 4000 years (Yamanoi, 1996). In contrast, 68 plots [referred to as forest (ski) plots] were located in areas where secondary forests or *Larix* plantations had been converted to ski courses during approximately 1965–1996 (Figure 2). We estimated the time since forest ski slopes were constructed through the clearcutting of developed forests using the aerial photographs. We calculated the duration from the first year that grassland was confirmed on each plot to 2019 (referred to as the durations of management; 10, 29, 45, 55, and 72 years).

### 2.4 Quantifying the pasture‐origin grassland area

To evaluate the quantity of potential seed supply sources, we utilized QGIS, a geographic information system software (QGIS Development Team, 2024), along with historical land‐use data to quantify the area of ski grasslands originating from pasture surrounding each survey plot. Initially, we identified areas where grasslands have been continuously managed from the time they were traditional pastures up to the present without undergoing forestation (Inoue et al., 2021). For each study site, we generated circles with radii of 50, 100, and 200 m from the center of the site. We then calculated the area of grasslands (in square meters) within these circles, considering potential range of seed sources for the study site. We did not calculate those of larger spatial scales to avoid significant overlaps among the circles.

### 2.5 Data analyses

In our data analyses, we utilized generalized linear models (GLMs) to examine the effect of past forest use on two diversity indices: species richness and Shannon's diversity index (*H*′) for all species, as well as for native and exotic grassland species and forest species, incorporating these metrics as response variables with Poisson distribution and log‐link function and Gaussian distribution and identity‐link function, respectively. The *H′* was defined as follows:

$$H^{\prime }=-\sum_{i=1}^{N}\left(\frac{a_{i}}{A}\right)\ln\left(\frac{a_{i}}{A}\right)$$

where N is the number of species within the plot, A is the total abundance of all species, and $a_{i}$ is the abundance of species i.

We also utilized GLMs to examine the effects of grassland management duration and area of surrounding seed source on diversity indices for all species, as well as for native and exotic grassland species and forest species, incorporating these indices as response variables with Poisson distribution and log‐link function and Gaussian distribution and identity‐link function, respectively. Furthermore, considering that the impact on the recovering process is expected to vary depending on the differences in seed dispersal modes among native grassland species, we calculated richness and the *H′* of the grassland species for each of six seed dispersal modes [barochory (gravity dispersal), ballochory (ballistic dispersal), hydrochory (water dispersal), myrmecochory (ant dispersal), anemochory (wind dispersal), zoochory (animal dispersal, including both epizoochory and endozoochory)]. Because the hydrochorous species were quite few (maximum 1 species per plot), we excluded this group from the analyses. Given that epizoochorous and endozoochorous species are less common and both modes have similar dispersal distances (Thomson et al., 2011), we grouped them together as zoochorous species in this study. Each was set as the response variable in our model. On the other hand, the explanatory variables included duration of grassland management (DGM), the log_10_‐transformed area of pasture ski slopes within radii of 50, 100, or 200 m (APS‐50, APS‐100, or APS‐200), and vegetation height in the full models. We included vegetation height as a covariate in the model because this variable often has strong impact on grassland species richness (Nagata & Ushimaru, 2016). Additionally, to account for the effects of spatial proximity and pseudo replication within each 10 × 10 m quadrat on the statistical result, we calculated and incorporated spatial autocovariates (Dormann et al., 2007). The autocovariates of the response variables were calculated from the latitude and longitude of each study plot by adding a distance‐weighted function of neighboring response values, incorporating as explanatory variables in the models (Dormann et al., 2007).

Furthermore, model selection was performed based on the corrected Akaike information criterion (AIC_c_) to examine the effects of DGM and APS and to clarify the most influential spatial scale (50, 100, or 200 m) from seed source. The model with the smallest AIC_c_ value was considered the best model, and models with ΔAIC_c_ ≤2 were also selected as the plausible models. Variables included in the best models are considered to have effects on each response variable. In model selection, decisions were made not only based on identifying the best model but also considering models with a ΔAIC_c_ (the difference in AIC_c_ between that model and the best model) of 2.0 or less. The subsequent plausible models are shown in Tables S1–S4 to present our analytical results in detail, although our discussion mostly based on the best models. During the model selection process, models that did not incorporate spatial autocorrelation were evaluated but ultimately excluded from the results due to their lack of consideration for the spatial similarity among response variables. We used R v4.2.2 and MuMIn package v1.47.5 (R core Team, 2016) for all these analyses.

## 3 RESULTS

### 3.1 Vegetation survey

In the study of 100 plots on ski slopes, we identified 139 plant species (Table S5), comprising 91 native and 17 exotic grassland species, and 31 native forest species. Regarding life‐history traits, we categorized 7 annual, 3 biennial, and 81 perennial species; the remaining were woody species. In terms of dispersal modes, 42 barochorous, 3 ballochorous, 1 hydrochorous, 6 myrmecochorous, 30 anemochorous, 2 epizoochorous, and 7 endozoochorous grassland species were observed.

### 3.2 GLM and model selection

According to the best model for comparing pasture and forest slopes, species richness and *H′* of both total and native grassland species decreased with past forest use, while species richness of exotic grassland and forest species increased with past forest use, while their *H′* did not vary between the slope types (Table 1, Table S1).

**TABLE 1:** Estimated coefficients for each explanatory variable in the best generalized linear models (GLMs), which were selected based on the corrected Akaike information criterion (AIC_c_), for species richness and *H′* of total species, as well as native and exotic grassland species and forest species, are provided.

Response variables	Estimated coefficient		
	Past forest use	Autocovariate	Intercept
Species richness of			
Total spp.	**−0.121 (−0.230, −0.102)**	**8.43 × 10** ^**−7**^ **(4.60 × 10** ^**−7**^ **, 12.2 × 10** ^**−7**^ **)**	**2.27 (2.05, 2.50)**
Native grassland spp.	**−0.273 (−0.401, −0.144)**	**1.33 × 10** ^**−6**^ **(7.88 × 10** ^**−7**^ **, 18.6 × 10** ^**−7**^ **)**	**2.00 (1.72, 2.28)**
Exotic grassland spp.	**0.809 (0.253, 1.43)**	**1.12 × 10** ^**−5**^ **(8.18 × 10** ^**−6**^ **, 1.41 × 10** ^**−5**^ **)**	**−1.08 (−1.64, −0.598)**
Forest spp.	0.422 (−0.0551, 0.932)	**9.53 × 10** ^**−6**^ **(6.62 × 10** ^**−6**^ **, 1.24 × 10** ^**−5**^ **)**	**−0.610 (−1.06, −0.217)**
*H*′ of			
Total spp.	−0.104 (−0.236, 0.0288)	1.35 × 10^−6^ (−1.85 × 10^−6^, 4.55 × 10^−6^)	**2.25 (1.96, 2.54)**
Native grassland spp.	**−0.387 (−0.537, −0.237)**	3.91 × 10^−6^ (−5.71 × 10^−8^, 7.87 × 10^−6^)	**1.97 (1.63, 2.31)**
Exotic grassland spp.		**2.78 × 10** ^**−5**^ **(2.25 × 10** ^**−5**^ **, 3.32 × 10** ^**−5**^ **)**	−0.0334 (−0.152, 0.0854)
Forest spp.		**2.23 × 10** ^**−5**^ **(1.73 × 10** ^**−5**^ **, 2.73 × 10** ^**−5**^ **)**	0.0511 (−0.0594, 0.162)

*Note*: The comprehensive models incorporate variables such as past forest use and an autocovariate. Values are presented as mean (95% confidence interval). Coefficients with 95% CIs not including zero are shown in bold. Details of subsequent models are provided in Table S1.

**FIGURE 3:**
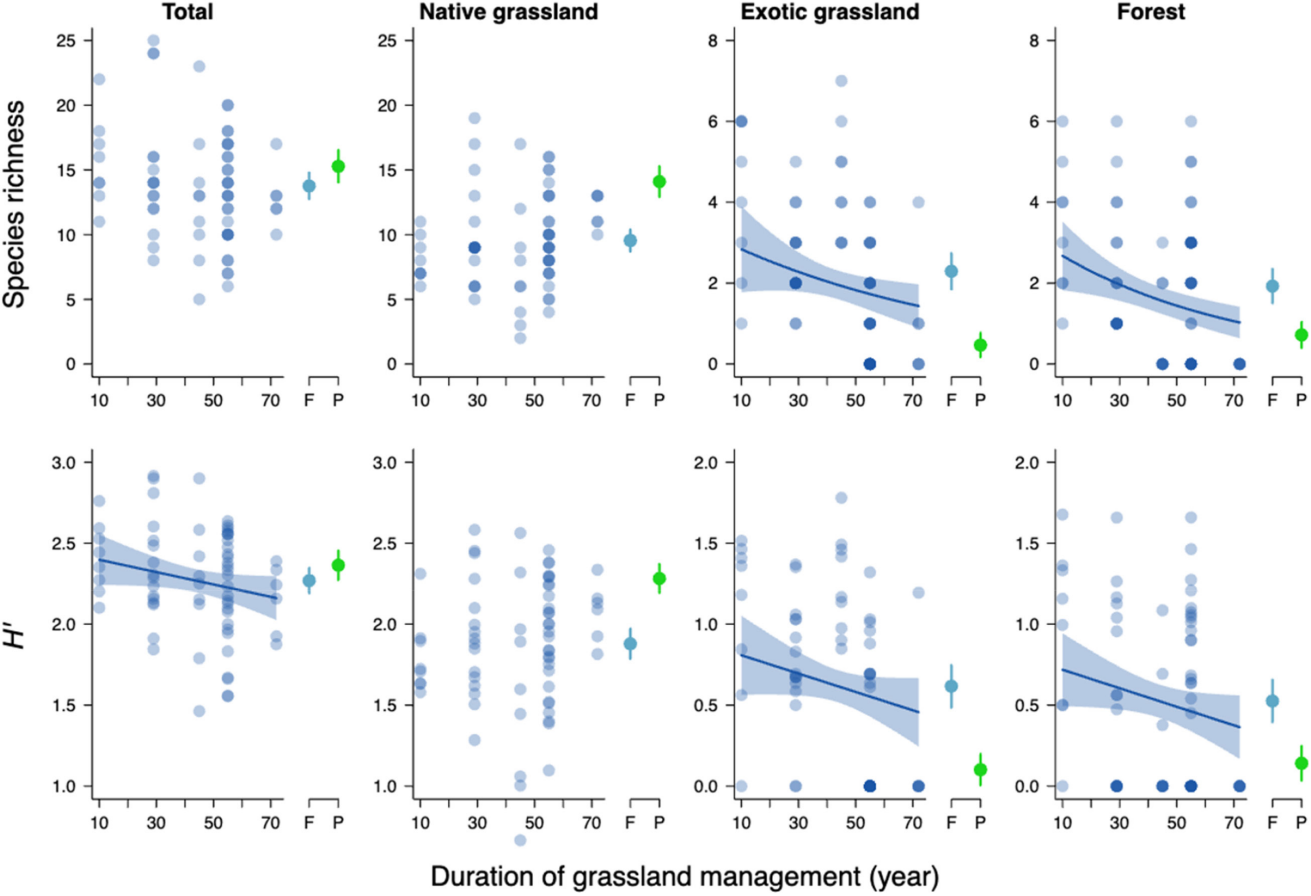
Relationship between the duration of grassland management (DGM, year) and diversity indices (the species richness and *H′*). Blue circles represent forest plots, and blue lines indicate regressions estimated in the best GLM with 95% confidence intervals (CIs). When DGM was not included in the best model, lines are not drawn. On the right side, blue and green circles indicate the means of diversity indices for forest (F) and pasture (P) plots, respectively, with error bars indicating 95% CIs.

According to the best model, the species richness of total species decreased with increasing vegetation height, while the *H′* of the total species decreased with DGM (Table 2, Table S2, Figure 3). Both the species richness and *H′* of the total species increased with APS‐100 (Table 2, Table S2, Figure S1). Similarly, the species richness and *H′* of native grassland species increased with APS‐100 (Table 2, Table S2, Figure S1). In contrast, the species richness and *H′* of exotic grassland and forest species decreased with DGM (Table 2, Table S2, Figure 3). The species richness and *H′* of exotic grassland species increased with APS‐200 and APS‐50, respectively whereas those of forest species were not influenced by APS variables (Table 2, Table S2, Figure 3).

**TABLE 2:** Estimated coefficients for each explanatory variable in the best generalized linear models (GLMs), which were selected based on the corrected Akaike information criterion (AIC_c_), for species richness and *H′* of total species, as well as native and exotic grassland species, are provided.

Response variables	Estimated coefficient				
	DGM	APS	VH	Autocovariate	Intercept
Species richness of					
Total spp.		^2^ **0.0758 (0.0253, 0.125)**	−0.00274 (−0.00602, 0.000489)	**5.87 × 10** ^**−7**^ **(1.40 × 10** ^**−7**^ **, 1.03 × 10** ^**−6**^ **)**	**2.42 (2.09, 2.74)**
Native grassland spp.		^2^ **0.0710 (0.0117, 0.128)**		**1.33 × 10** ^**−6**^ **(6.87 × 10** ^**−7**^ **, 1.97 × 10** ^**−6**^ **)**	**1.73 (1.48, 1.98)**
Exotic grassland spp.	**−0.0110 (−0.0216, −0.000371)**	^3^0.0849 (−0.00879, 0.175)		**8.61 × 10** ^**−6**^ **(4.88 × 10** ^**−6**^ **, 1.23 × 10** ^**−5**^ **)**	0.344 (−0.366, 1.03)
Forest spp.	**−0.01545 (−0.0247, −0.00607)**			**9.60 × 10** ^**−6**^ **(6.42 × 10** ^**−6**^ **, 1.28 × 10** ^**−5**^ **)**	0.436 (−0.0674, 0.927)
*H′* of					
Total spp.	−0.00382 (−0.00790, 0.000270)	^2^ **0.102 (0.0411, 0.162)**		2.23 × 10^−6^ (−1.08 × 10^−6^, 5.54 × 10^−6^)	**2.20 (1.86, 2.55)**
Native grassland spp.		^2^ **0.0945 (0.0411, 0.162)**		4.81 × 10^−6^ (−1.69 × 10^−7^, 9.10 × 10^−6^)	**1.50 (1.03, 1.79)**
Exotic grassland spp.	−0.00567 (−0.0123, 0.000946)	^1^0.0796 (−0.0254, 0.185)		**2.18 × 10** ^**−5**^ **(1.27 × 10** ^**−5**^ **, 3.08 × 10** ^**−5**^ **)**	0.345 (−0.0941, 0.784)
Forest spp.	−0.00572 (−0.0116, 0.000209)			**2.01 × 10** ^**−5**^ **(1.37 × 10** ^**−5**^ **, 2.66 × 10** ^**−5**^ **)**	**0.36880 (0.0427, 0.695)**

*Note*: The comprehensive models incorporate variables such as duration of grassland management (DGM), log_10_‐transformed area of pasture ski slopes (APS) within radii of 50, 100, and 200 m (denoted as 1, 2, and 3, respectively, using left superscript), vegetation height (VH), and autocovariate. Values are presented as mean (95% confidence interval, CI). Coefficients with 95% CIs not including zero are shown in bold. Details of subsequent models are provided in Table S2.

In terms of dispersal modes, the best model for comparing pasture and forest slopes, both species richness and *H′* of barochorous and anemochorous grassland species decreased with past forest use (Table S3), while both species richness and *H′* of ballochorous, myrmecochorous, and zoochorous grassland species did not vary between the slope types (Table S3).

**FIGURE 4:**
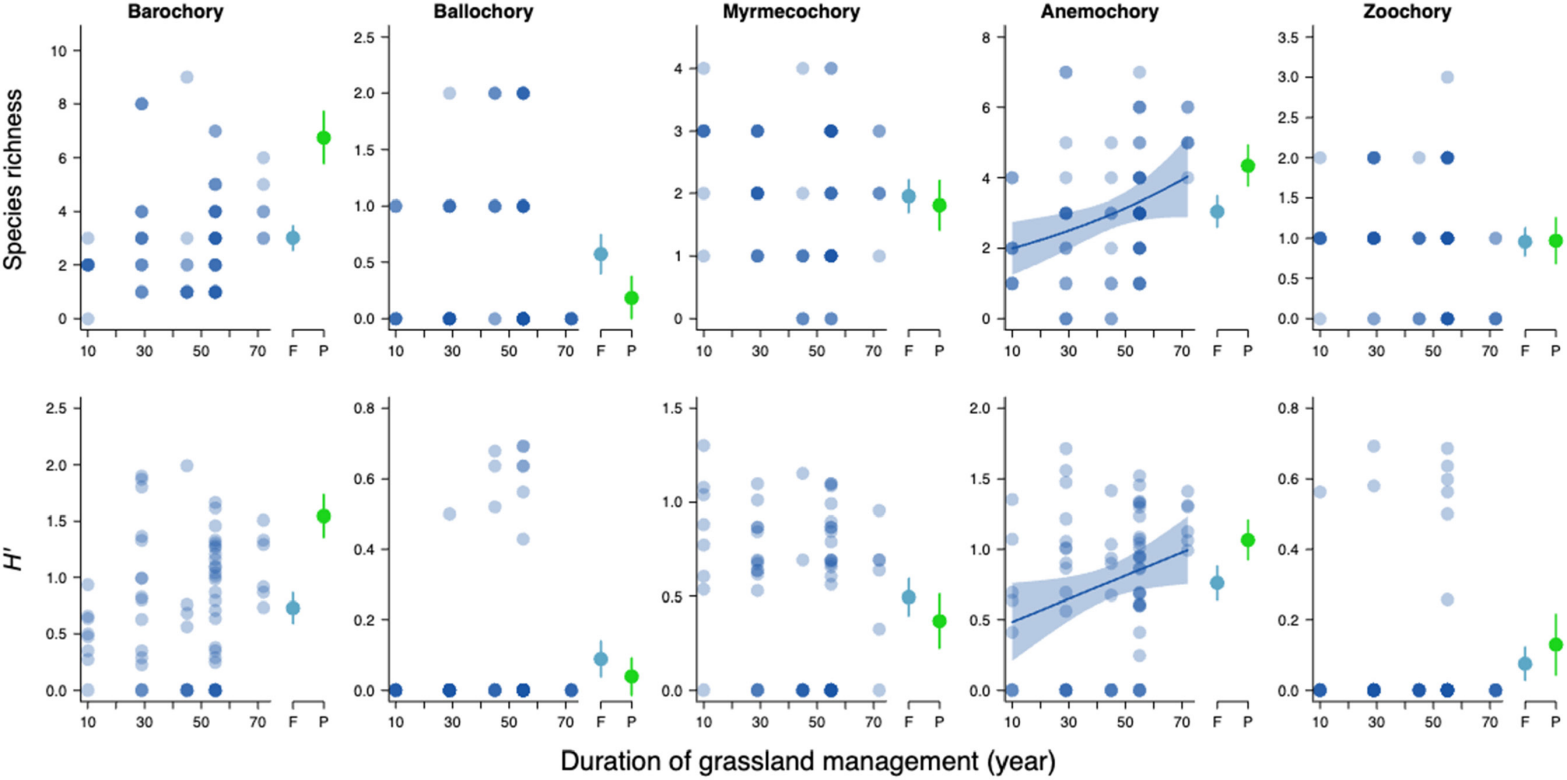
Relationship between the duration of grassland management (DGM, year) and diversity indices (the species richness and *H′*) of native grassland species, categorized by dispersal modes: barochory, ballochory, myrmecochory, anemochory, and zoochory. Blue circles represent forest plots, and blue lines indicate regressions estimated in the best GLM with 95% confidence intervals (CIs). When DGM was not included in the best model, lines are not drawn. On the right side, blue and green circles indicate the means of diversity indices for forest (F) and pasture (P) plots, respectively, with error bars indicating 95% CIs.

The best models for richness of barochorous grassland species did not include DGM and APS, which means that only the autocovariate was included (Table 3). Meanwhile, the best model indicated that their *H′* increased with APS‐100 (Table 3, Table S4, Figure S2). According to the best models, richness of ballochorous grassland species did not vary with both DGM and APS, while their *H′* increased with APS‐200 (Table 3, Table S4). The best model indicated that richness and *H′* of myrmecochorous grassland species decreased with vegetation height (Table 3, Table S4). Both species richness and the *H′* of anemochorous grassland species increased with DGM and APS‐100 (Table 3, Table S4, Figure 4, Figure S2). Lastly, the best model indicated that species richness and *H′* of zoochorous grassland species did not vary with DGM and APS (Table 3, Table S4).

**TABLE 3:** Estimated coefficients for each explanatory variable in the best generalized linear models (GLMs), which were selected based on the corrected Akaike information criterion (AIC_c_), for species richness and *H′* of native grassland species, categorized as barochorous, ballochorous, hydrochorous, myrmecochorous, anemochorous, and zoochorous, are provided.

Response variables	Estimated coefficient				
	DGM	APS	VH	Autocovariate	Intercept
Richness of					
Barochorous grassland spp.				**7.34 × 10** ^**−6**^ **(5.32 × 10** ^**−6**^ **, 9.34 × 10** ^**−6**^ **)**	0.1580 (−0.170, 0.473)
Ballochorous grassland spp.				**3.23 × 10** ^**−5**^ **(1.50 × 10** ^**−5**^ **, 4.96 × 10** ^**−5**^ **)**	**−1.42 (−2.10, −0.827)**
Myrmecochorous grassland spp.			**−0.00883 (−0.0180, −8.69 × 10** ^**−5**^ **)**	5.34 × 10^−6^ (−8.88 × 10^−7^, 1.14 × 10^−5^)	0.714 (−0.0300, 1.44)
Anemochorous grassland spp.	**0.0114 (0.00186, 0.0212)**	^2^0.106 (−0.00578, 0.209)		2.26 × 10^−6^ (−1.19 × 10^−6^, 5.60 × 10^−6^)	0.278 (−0.219, 0.748)
Zoochorous grassland spp.				9.25 × 10^−6^ (−2.97 × 10^−7^, 1.75 × 10^−5^)	−0.406 (−0.856, 0.0352)
*H′* of					
Barochorous grassland spp.		^2^0.0681 (−0.0183, 0.155)		**2.30 × 10** ^**−5**^ **(1.69 × 10** ^**−5**^ **, 2.91 × 10** ^**−5**^ **)**	0.540 (−0.141, 0.249)
Ballochorous grassland spp.		^3^0.0309 (−0.000586, 0.0625)		9.33 × 10^−6^ (−2.44 × 10^−6^, 2.11 × 10^−5^)	0.0237 (−0.0414, 0.0888)
Myrmecochorous grassland spp.			**−0.00545 (−0.0101, −0.000794)**	8.08 × 10^−6^ (−3.23 × 10^−6^, 1.94 × 10^−5^)	**0.636 (0.251, 1.02)**
Anemochorous grassland spp.	**0.00824 (0.000658, 0.0158)**	^2^0.0925 (−0.0121, 0.197)		−1.12 × 10^−6^ (−1.36 × 10^−5^, 1.14 × 10^−5^)	**0.393 (0.00356, 0.781)**
Zoochorous grassland spp.				1.597 × 10^−5^ (−5.01 × 10^−6^, 1.49 × 10^−5^)	**0.0596 (0.00285, 0.116)**

*Note*: The comprehensive models incorporate variables such as duration of grassland management (DGM), log_10_‐transformed area of pasture ski slopes (APS) within radii of 100 and 200 m (denoted as 2 and 3, respectively, using left superscript), vegetation height (VH), and autocovariate. Values are presented as mean (95% confidence interval, CI). Coefficients with 95% CIs not including zero are shown in bold. Details of subsequent models are provided in Table S4.

## 4 DISCUSSION

We found that both the proximity to neighboring seed source grasslands and/or continuous, long‐term grassland management are important for restoring native grassland plants, particularly those with gravity‐ and wind‐dispersed seeds. These findings partially support the seed dispersal limitation hypothesis (Cousins & Lindborg, 2008; Matsumura & Takeda, 2010), which explains the negative legacy effect on the restoration process of grassland plants. The recovery of species with these two major dispersal modes, gravity and wind dispersal, was more pronounced in areas close to the seed source grasslands. Furthermore, the restoration of wind‐dispersed species was found to be dependent on the duration of grassland management in ski resorts. The results align with our expected outcomes.

Our findings also showed that the proximity of pasture slopes, such as within a radius of 100 m, to each study plot is more important than the long‐term grassland management for recovering the entire diversity of native grassland plants. This result highlighted that the area of adjacent species‐rich grasslands is especially crucial as an important factor in species restoration. Also, focusing on two main dispersal modes, the area of surrounding pasture slopes (APS) is suggested to be important for restoring short dispersal (gravity‐dispersed) species in terms of their diversity (*H′*) and long dispersal (wind‐dispersed) species in terms of both their richness and diversity.

This study revealed that while continuous, long‐term grassland management is not effective for restoring the entire diversity of native grassland plants in forest ski slopes, it is particularly beneficial for grassland plants with wind‐dispersed seeds. For these species, a level of species richness and diversity equivalent to that found on pasture slopes was confirmed on forest slopes after more than 70 years of grassland management. Wind‐dispersed species, which have higher dispersal probability due to their relatively longer dispersal distance compared with gravity‐ and ballistic‐dispersed species, are more likely to successfully colonize annually mowed grassland over time. On the other hand, short‐distance dispersed species such as those with barochory did not recover with long‐term grassland management, even where neighboring seed source were present. These findings further support the seed dispersal limitation hypothesis, suggesting that a portion of the long‐term negative legacy effect of past afforestation on grassland vegetation can be explained by the limited dispersal abilities of certain species. Thus, in the light of the negative legacy effects hindering vegetation restoration, this study not only reinforces the importance of long‐term grassland management and surrounding seed sources, as highlighted in previous studies (Cousins & Lindborg, 2008; Lindborg & Eriksson, 2004; Matsumura & Takeda, 2010), but also difference in restoration process between the major two dispersal modes.

The reason why the diversity of all species decreased with long‐term grassland management over 70 years is the decrease in the diversity of exotic and forest species. This finding is particularly valuable when considering the problem where native grassland species are declining due to competition with exotic and forest species (Daehler, 2003; Vilà & Weiner, 2004). In practice, exotic forage species such as *Dactylis glomerata, Lolium arundinaceum* and *Trifolium* spp., were confirmed to dominate forest ski slopes by introducing through artificial seeding (Tsuyuzaki, 1993). These species were barochorous and rarely found in pasture ski slopes. Many forest plant species are often maintained on ski slopes created by clearing forests (Burt & Rice, 2009; Inoue et al., 2021). Our results suggest that maintaining grassland conditions by continuous annual mowing has excluded the abundant trees and shrubs, which are abundant in surrounding forests, in forest slopes. The dominance of exotic and forest species in the initial restored grasslands (forest slopes in this study) is an aspect of the negative legacy effect which has not been considered before, but it is important for restoring native grassland species through reintroducing grassland management practices. Our finding suggests that long‐term ski slope management through annual mowing has mitigated the negative impact of artificial seeding of exotic species and forest species invasion on the restoration of native grassland vegetation.

For species groups with ballochory (self‐dispersal by mechanisms like explosion) and zoochory (including epizoochory and endozoochory), the null models were the best models of both species richness and *H′*, suggesting no seed dispersal limitations in their recoveries. Furthermore, richness and/or diversity of these groups on forest slopes were higher than or comparable to those on pasture slopes (Figure 4). Longer dispersal abilities of zoochorous species than anemochorous species likely supported their quick recovery in restored grasslands as we expected (Thomson et al., 2011). Meanwhile, higher richness and diversity of ballochorous species on forest slopes cannot be explained by seed dispersal limitation hypothesis. We found that richness and diversity of plant species with myrmecochory (seed dispersal by ants), such as *Arenaria lateriflora*, *Carex leucochlora*, and *Viola grypoceras*, on forest slopes were equivalent to those on pasture slopes and did not vary with DGM and APS (Table S3), suggestion no seed dispersal limitation in the group. Myrmecochorous species invaded earlier after forest clearance likely due to high activities of ants that prefer disturbed grounds (Arruda et al., 2020).

The richness and diversity native grassland species on forest slopes were significantly lower than those on pasture slopes, supporting the previous studies (Inoue et al., 2021; Yaida et al., 2022). These differences were not fully explained by the negative effects of machine grading and current environmental conditions (Yaida et al., 2022). This study further explains that seed dispersal limitations, while depending on their dispersal modes, were factors preventing native species from recovering. Overall, it has been confirmed that the area of the surrounding seed source grasslands and the duration of grassland management collectively had significant impacts on the species richness and diversity of these grassland plants. Therefore, when conserving and managing plant communities on forest ski slopes grasslands, it is necessary to comprehensively consider seed dispersal limitation in gravity‐ and wind‐dispersed seeds. Especially given the rapid recent decline of semi‐natural grasslands and plant diversity therein (Koyanagi & Furukawa, 2013), their conservation on forest ski slopes is an urgent issue in our country (Ushimaru et al., 2018). It may be necessary to actively sow seeds to restore grassland vegetation for species with short dispersal distances (Martin & Wilsey, 2006; Török et al., 2010). However, when sowing, it is essential to collect seeds from nearby grasslands to prevent genetic contamination. Furthermore, this study has also revealed that taller vegetation diminished total species richness on forest slopes. It is expected that controlling vegetation height during seeding will also lead to efficient vegetation restoration. This research suggests that on restored grasslands (forest ski slopes in our case), it is necessary to continue management over a long‐term timescale for grassland restoration and to properly conserve nearby species‐rich native grasslands as a seed source.

## AUTHOR CONTRIBUTIONS

**Yuki A. Yaida:** Conceptualization (lead); data curation (lead); formal analysis (lead); investigation (lead); methodology (lead); project administration (equal); resources (lead); software (lead); validation (lead); visualization (lead); writing – original draft (lead). **Taiki Inoue:** Conceptualization (supporting); data curation (supporting); writing – review and editing (supporting). **Tanaka Kenta:** Conceptualization (supporting); project administration (equal); validation (equal); writing – review and editing (supporting). **Atushi Ushimaru:** Conceptualization (equal); formal analysis (equal); methodology (equal); project administration (equal); supervision (lead); validation (equal); writing – original draft (equal); writing – review and editing (lead).

## CONFLICT OF INTEREST STATEMENT

The authors declare no conflict of interest.

### OPEN RESEARCH BADGES

This article has earned an Open Data badge for making publicly available the digitally‐shareable data necessary to reproduce the reported results.

## Supporting information


Data S1

## Data Availability

Data‐EcoEvo‐yaida‐2024.xlsx were row data to use in the analysis of our MS. These data included this URL (https://zenodo.org/records/10963543).
