# Supplementary material for: Seed dispersal limitation causes negative legacy effect on restoration of grassland plant diversity on ski slopes
Source: Ecol Evol. 2024 Jul 7;14(7):e11654. doi: 10.1002/ece3.11654 (PMC11227990; doi:10.1002/ece3.11654)
Supplement: Supplementary file 1 — Data S1 [file ECE3-14-e11654-s001.docx]

Table S1. Best and all plausible generalized linear models (GLMs) for species richness and Shannon’s diversity index (*H′*) of total species, as well as of native and exotic grassland species and forest species, were selected based on the corrected Akaike information criterion (AICc). ΔAICc indicates the difference in AICc between each model and the best model. The best model and the ΔAICc ≦ 2 subsequent all plausible models are displayed. Estimated coefficients for each explanatory variable across all models are provided. The comprehensive models included variables such as land-use history and autocovariate.

|  | Estimated coefficients | | |  |
| --- | --- | --- | --- | --- |
| Response variables | Past forest use | Autocovariate | Intercept | ΔAICc |
| Species richness of |  |  |  |  |
| total spp. | -1.274 | 8.433×10^-7^ | 2.274 | 0.00 |
|  |  |  |  |  |
| native grassland spp. | -0.2729 | 1.326×10^-6^ | 2.003 | 0.00 |
|  |  |  |  |  |
| exotic grassland spp. | 0.8087 | 1.117×10^-5^ | -1.075 | 0.00 |
|  |  |  |  |  |
| forest spp. | 0.422 | 9.527×10^-6^ | -0.6099 | 0.00 |
|  |  | 1.073×10^-5^ | -0.3732 | 0.86 |
| *H′* of |  |  |  |  |
| total spp. | -0.1039 | 1.351×10^-6^ | 2.251 | 0.00 |
|  |  | 9.603×10^-7^ | 2.214 | 0.29 |
|  |  |  |  |  |
| native grassland spp. | -0.3870 | 3.907×10^-6^ | 1.971 | 0.00 |
|  |  |  |  |  |
| exotic grassland spp. |  | 2.783×10^-5^ | -0.03338 | 0.00 |
|  | 0.06912 | 2.652×10^-5^ | -0.05762 | 1.65 |
|  |  |  |  |  |
| forest spp. |  | 2.227×10^-5^ | 0.05110 | 0.00 |
|  | 0.07467 | 2.127×10^-^ | 0.01603 | 1.51 |

Table S2. Best and all plausible generalized linear models (GLMs) for species richness and Shannon’s diversity index (*H′*) of total species, as well as of native and exotic grassland species and forest species, were selected based on the corrected Akaike information criterion (AICc). ΔAICc indicates the difference in AICc between each model and the best model. The best model and the ΔAICc ≦ 2 subsequent all plausible models are displayed. Estimated coefficients for each explanatory variable across all models are provided. The comprehensive models included variables such as duration of grassland management (DGM), log10-transformed area of pasture ski slopes (APS) within radii of 50 m, 100 m, and 200 m (denoted as -50, -100, and -200, respectively), vegetation height (VH), and autocovariate.

|  | Estimated coefficients | | | | | | |  |
| --- | --- | --- | --- | --- | --- | --- | --- | --- |
| Response variables | DGM | APS-50 | APS-100 | APS-200 | VH | Autocovariate | Intercept | ΔAICc |
| Species richness of |  |  |  |  |  |  |  |  |
| total spp. |  |  | 0.07576 |  | -0.002740 | 5.866×10^-7^ | 2.417 | 0.00 |
|  |  |  | 0.08472 |  |  | 6.855×10^-7^ | 2.220 | 0.50 |
|  | -0.002807 |  | 0.08072 |  |  | 6.654×10^-7^ | 2.353 | 0.56 |
|  |  | 0.08671 |  |  | -0.002838 | 6.125×10^-7^ | 2.411 | 0.89 |
|  | -0.002003 |  | 0.07472 |  | -0.002182 | 5.928×10^-7^ | 2.472 | 1.32 |
|  | -0.002889 | 0.09291 |  |  |  | 6.956×10^-7^ | 2.343 | 1.52 |
|  |  | 0.09738 |  |  |  | 7.179×10^-7^ | 2.205 | 1.60 |
|  |  |  |  |  |  |  |  |  |
| native grassland spp. |  |  | 0.07095 |  |  | 1.330×10^-6^ | 1.731 | 0.00 |
|  |  | 0.08096 |  |  |  | 1.363×10^-6^ | 1.721 | 0.68 |
|  | 0.002904 |  | 0.07894 |  |  | 1.234×10^-6^ | 1.633 | 0.80 |
|  | 0.002700 | 0.08909 |  |  |  | 1.277×10^-6^ | 1.630 | 1.67 |
|  | 0.003907 |  | 0.07220 |  | -0.002340 | 1.132×10^-6^ | 1.751 | 1.85 |
|  |  |  | 0.06588 |  | -0.001235 | 1.295×10^-6^ | 1.810 | 1.85 |
|  |  |  |  |  |  |  |  |  |
| exotic grassland spp. | -0.01100 |  |  | 0.08490 |  | 8.613×10^-6^ | 0.3444 | 0.00 |
|  | -0.009316 | 0.1244 |  |  |  | 8.496×10^-6^ | 0.3288 | 0.01 |
|  | -0.009051 |  | 0.1005 |  |  | 8.593×10^-6^ | 0.3109 | 0.19 |
|  |  | 0.1110 |  |  |  | 1.025×10^-5^ | -0.2077 | 0.89 |
|  | -0.008227 |  |  |  |  | 8.887×10^-6^ | 0.3088 | 0.91 |
|  |  |  | 0.09265 |  |  | 1.029×10^-5^ | -0.2114 | 0.91 |
|  |  |  |  |  |  | 1.046×10^-5^ | -0.1734 | 1.26 |
|  |  |  |  |  | -0.005434 | 9.816×10^-6^ | 0.1588 | 1.83 |
|  |  |  |  | 0.05807 |  | 1.064×10^-5^ | -0.2610 | 1.85 |
|  | -0.009877 |  |  | 0.07943 | -0.003056 | 8.458×10^-6^ | 0.4728 | 1.86 |
|  | -0.008395 | 0.1157 |  |  | -0.002779 | 8.369×10^-6^ | 0.4465 | 1.96 |
|  |  |  |  |  |  |  |  |  |
| *H′* of |  |  |  |  |  |  |  |  |
| total spp. | -0.003815 |  | 0.10180 |  |  | 2.226×10^-6^ | 2.202 | 0.00 |
|  |  |  | 0.09600 |  | -0.002741 | 1.781×10^-6^ | 2.220 | 0.84 |
|  | -0.003043 |  | 0.09468 |  | -0.001923 | 1.932×10^-6^ | 2.298 | 1.15 |
|  | -0.003916 | 0.1161 |  |  |  | 2.270×10^-6^ | 2.205 | 1.25 |
|  |  |  | 0.10780 |  |  | 2.190×10^-6^ | 2.036 | 1.27 |
|  |  | 0.1087 |  |  | -0.002874 | 1.802×10^-6^ | 2.228 | 1.98 |
|  |  |  |  |  |  |  |  |  |
| native grassland spp. |  |  | 0.09447 |  |  | 4.810×10^-6^ | 1.498 | 0.00 |
|  |  | 0.1057 |  |  |  | 4.877×10^-6^ | 1.497 | 0.92 |
|  | 0.002484 |  | 0.09845 |  |  | 4.466×10^-6^ | 1.412 | 1.36 |
|  |  |  |  |  |  |  |  |  |
| exotic grassland spp. | -0.005665 | 0.07964 |  |  |  | 2.177×10^-5^ | 0.3451 | 0.00 |
|  | -0.005415 |  |  |  |  | 2.352×10^-5^ | 0.3231 | 0.06 |
|  | -0.005569 |  | 0.06238 |  |  | 2.197×10^-5^ | 0.3379 | 0.24 |
|  |  |  |  |  |  | 2.727×10^-5^ | 0.001711 | 0.50 |
|  |  | 0.07515 |  |  |  | 2.577×10^-5^ | 0.008481 | 0.71 |
|  |  |  | 0.06001 |  |  | 2.588×10^-5^ | 0.007125 | 0.85 |
|  |  |  |  |  | -0.002963 | 2.563×10^-5^ | 0.194900 | 1.20 |
|  | -0.006410 |  |  | 0.03155 |  | 2.238×10^-5^ | 0.358800 | 1.33 |
|  | -0.004767 |  |  |  |  | 2.272×10^-5^ | 0.432100 | 1.49 |
|  | -0.005148 | 0.07386 |  |  |  | 2.128×10^-5^ | 0.427400 | 1.87 |
|  |  | 0.06731 |  |  |  | 2.452×10^-5^ | 0.173400 | 1.89 |


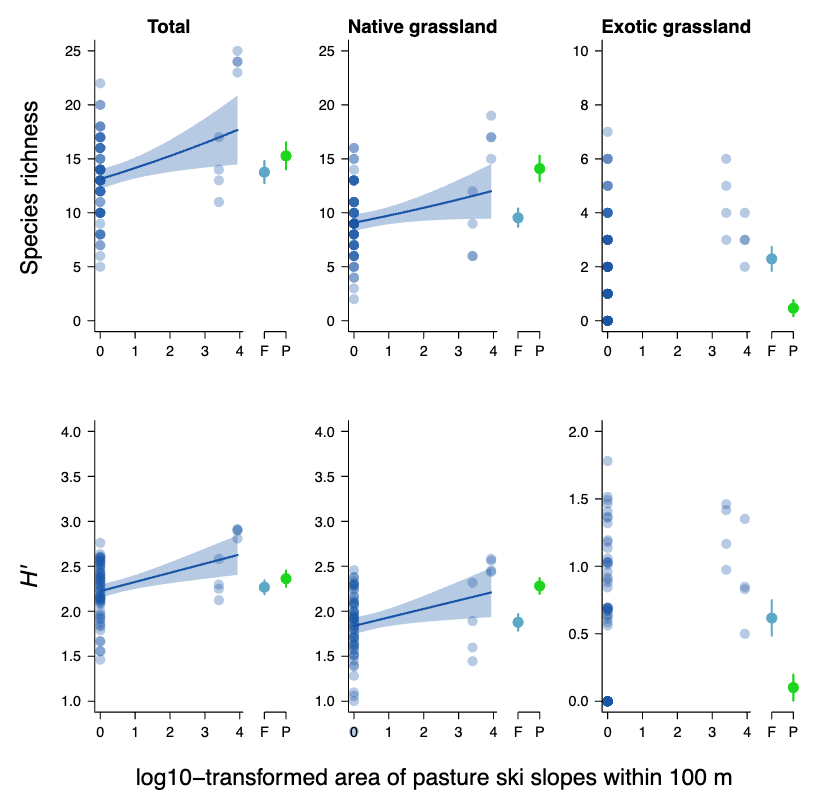


Fig. S1. Relationship between log_10_-transformed area of pasture ski slopes within a 100-m radius (APS-100) and diversity indices (the species richness and *H′*). Blue circles represent forest plots, and blue lines indicate regressions estimated in the best GLM with 95% confidence intervals (CIs). When DGM was not included in the best model, lines are not drawn. On the right side, blue and green circles indicate the means of diversity indices for forest (F) and pasture (P) plots, respectively, with error bars indicating 95% CIs.

Table S3. Best and all plausible generalized linear models (GLMs) for species richness and *H′* of native grassland species, categorized as barochorous, ballochorous, myrmecochorous, anemochorous, and zoochorous (including both endozoochorous and epizoochorous), were selected based on the corrected Akaike information criterion (AICc). ΔAICc indicates the difference in AICc between each model and the best model. The best model and the ΔAICc ≦ 2 subsequent all plausible models are displayed. Estimated coefficients for each explanatory variable across all models are provided. The comprehensive models included variables such as land-use history and autocovariate.

|  | Estimated coefficients | | |  |
| --- | --- | --- | --- | --- |
| Response variables | Past forest use | Autocovariate | Intercept | ΔAICc |
| Species richness of |  |  |  |  |
| barochorous grassland spp. | -0.3061 | 5.282×10^-6^ | 0.6719 | 0.00 |
|  |  |  |  |  |
| ballochorous grassland spp. |  | 4.115×10^-5^ | -1.821 | 0.00 |
|  | 0.5127 | 3.795×10^-5^ | -2.147 | 0.75 |
|  |  |  |  |  |
| myrmecochorous grassland spp. |  | 7.923×10^-6^ | 0.05539 | 0.00 |
|  |  |  |  |  |
| anemochorous grassland spp. | -0.2162 | 4.494×10^-6^ | 0.7636 | 0.00 |
|  |  | 5.119×10^-6^ | 0.5395 | 1.36 |
|  |  |  |  |  |
| zoochorous grassland spp. |  | 1.281×10^-5^ | -0.5583 | 0.00 |
|  | -0.09507 | 1.303×10^-5^ | -0.5027 | 1.94 |
|  |  |  |  |  |
| *H′* of |  |  |  |  |
| barochorous grassland spp. | -0.3600 | 2.236×10^-5^ | 0.3896 | 0.00 |
|  |  |  |  |  |
| ballochorous grassland spp. |  | 1.630×10^-5^ | 0.02470 | 0.00 |
|  | 0.02120 | 1.583×10^-5^ | 0.01164 | 1.90 |
|  |  |  |  |  |
| myrmecochorous grassland spp. |  | 1.632×10^-5^ | 0.1782 | 0.00 |
|  |  |  |  |  |
| anemochorous grassland spp. | -0.2323 | 1.054×10^-5^ | 0.6757 | 0.00 |
|  |  |  |  |  |
| zoochorous grassland spp. |  | 7.880×10^-6^ | 0.06349 | 0.00 |
|  | -0.04851 | 7.507×10^-6^ | 0.09783 | 1.03 |

Table S4. Best and all plausible generalized linear models (GLMs) for specie richness and *H′* of native grassland species, categorized as barochorous, ballochorous, myrmecochorous, anemochorous, and zoochorous (including both endozoochorous and epizoochorous), were selected based on the corrected Akaike information criterion (AICc). ΔAICc indicates the difference in AICc between each model and the best model. The best model and the ΔAICc ≦ 2 subsequent all plausible models are displayed. Estimated coefficients for each explanatory variable across all models are provided. The comprehensive models included variables such as duration of grassland management (DGM), log10-transformed area of pasture ski slopes (APS) within radii of 50 m, 100 m, and 200 m (denoted as -50, -100, and -200, respectively), vegetation height, and autocovariate.

|  | Estimated coefficient | | | | | | |  |
| --- | --- | --- | --- | --- | --- | --- | --- | --- |
| Response variables | DGM | APS-50 | APS-100 | APS-200 | Plant height | Autocovariate | Intercept | ΔAICc |
| Species richness of |  |  |  |  |  |  |  |  |
| barochorous grassland spp. |  |  |  |  |  | 7.343×10^-6^ | 0.1580 | 0.00 |
|  | 0.003318 |  |  |  |  | 7.333×10^-6^ | 0.009968 | 1.54 |
|  |  |  | 0.02516 |  |  | 7.048×10^-6^ | 0.1823 | 1.98 |
|  |  |  |  |  |  |  |  |  |
| ballochorous grassland spp. |  |  |  |  |  | 3.226×10^-5^ | -1.423 | 0.00 |
|  |  |  |  |  | 0.008683 | 3.428×10^-5^ | -1.953 | 0.91 |
|  |  |  |  | 0.09329 |  | 3.025×10^-5^ | -1.489 | 1.20 |
|  | -0.006086 |  |  |  |  | 3.370×10^-5^ | -1.196 | 1.87 |
|  |  |  |  |  |  |  |  |  |
| myrmecochorous grassland spp. |  |  |  |  | -0.008832 | 5.336×10^-6^ | 0.7137 | 0.00 |
|  |  |  |  |  |  | 6.925×10^-6^ | 0.1507 | 1.73 |
|  |  |  |  |  |  |  |  |  |
| anemochorous grassland spp. | 0.01135 |  | 0.1055 |  |  | 2.258×10^-6^ | 0.2782 | 0.00 |
|  | 0.01097 | 0.1187 |  |  |  | 2.397×10^-6^ | 0.2834 | 0.44 |
|  | 0.008873 |  |  |  |  | 2.951×10^-6^ | 0.3641 | 1.21 |
|  |  |  |  |  |  |  |  |  |
| zoochorous grassland sp. |  |  |  |  |  | 9.252×10^-6^ | -0.4057 | 0.00 |
|  |  |  |  |  | -0.008780 | 1.034×10^-5^ | -0.001447 | 0.20 |
|  | -0.007343 |  |  |  |  | 9.392×10^-6^ | -0.09989 | 1.10 |
|  |  |  |  | -0.04395 |  | 8.436×10^-6^ | -0.3321 | 1.91 |
|  |  |  |  |  |  |  |  |  |
| *H′* of |  |  |  |  |  |  |  |  |
| barochorous grassland spp. |  |  | 0.06811 |  |  | 2.298×10^-5^ | 0.05401 | 0.00 |
|  |  |  |  |  |  | 2.403×10^-5^ | 0.05382 | 0.29 |
|  |  | 0.07410 |  |  |  | 2.324×10^-5^ | 0.04956 | 0.45 |
|  |  |  |  | 0.02745 |  | 2.363×10^-5^ | 0.03610 | 1.71 |
|  |  |  |  |  |  |  |  |  |
| ballochorous grassland spp. |  |  |  | 0.03094 |  | 9.330×10^-6^ | 0.02368 | 0.00 |
|  |  |  |  | 0.03147 | 0.001044 | 9.530×10^-6^ | -0.03257 | 1.42 |
|  |  |  |  |  |  | 1.334×10^-6^ | 0.04310 | 1.64 |
|  |  |  |  |  |  |  |  |  |
| myrmecochorous grassland spp. |  |  |  |  | -0.005446 | 8.083×10^-6^ | 0. 6358 | 0.00 |
|  | -0.001743 |  |  |  | -0.005049 | 7.616×10^-6^ | 0.6996 | 1.96 |
|  |  |  |  |  |  |  |  |  |
| anemochorous grassland spp. | 0.008241 |  | 0.0925 |  |  | -1.121×10^-6^ | 0.3925 | 0.00 |
|  | 0.008037 | 0.1026 |  |  |  | -7.167×10^-7^ | 0.3932 | 0.48 |
|  | 0.007025 |  |  |  |  | 1.153×10^-6^ | 0.4220 | 0.90 |
|  | 0.007279 |  | 0.1008 |  | 0.002336 | -1.007×10^-6^ | 0.3047 | 1.74 |
|  |  |  |  |  |  |  |  |  |
| zoochorous grassland spp. |  |  |  |  |  | 4.925×10^-6^ | 0.05959 | 0.00 |
|  |  |  |  | -0.01546 |  | 3.488×10^-6^ | 0.08044 | 1.13 |
|  |  | -0.02379 |  |  |  | 3.928×10^-6^ | 0.07117 | 1.32 |
|  |  |  | -0.01963 |  |  | 3.928×10^-6^ | 0.07111 | 1.33 |
|  |  |  |  |  | -0.000644 | 5.106×10^-6^ | 0.09298 | 1.89 |


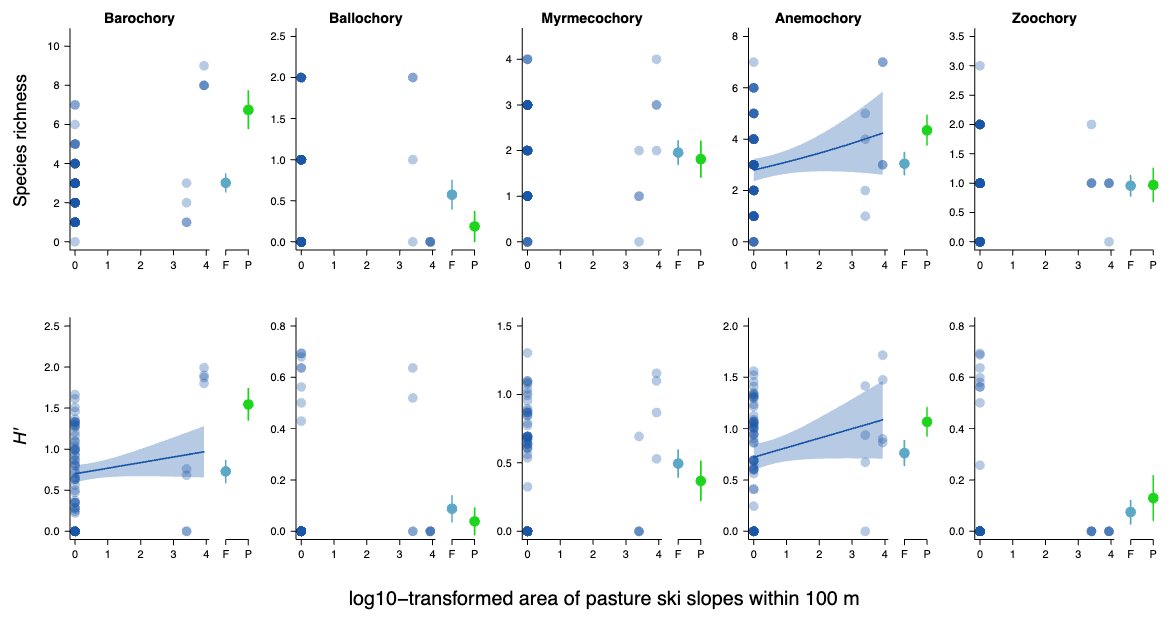


Fig. S2. Relationship between log_10_-transformed area of pasture ski slopes within a 100-m radius (APS-100) and diversity indices (species richness and *H′*) of native grassland species, categorized by dispersal modes: barochory, ballochory, hydrochory, myrmecochory, anemochory, and zoochory (including epizoochory and endozoochory). Blue circles represent forest plots, and blue lines indicate regressions estimated from the best GLM with 95% confidence intervals (CIs). When DGM was not included in the best model, lines are not drawn. On the right side, blue and green circles indicate the means of diversity indices for forest (F) and pasture (P) plots, respectively, with error bars indicating 95% CIs.

Table S5. Information on species detected in the study plots: origin (Native vs. Exotic); habitat (grassland species vs. forest and forest floor species, G/F); dispersal mode (Barochory, Ballochory, Hydrochory, Myrmecochory, Anemochory, Epizoochory, and Endozoochory).

| **Family Species** | | **Origin** | **Habitat** | **Dispersal mode** |
| --- | --- | --- | --- | --- |
| *Anacardiaceae* | |  |  |  |
|  | *Toxicodendron orientale* | Native | F | Endozoochory |
| *Apiaceae* | |  |  |  |
|  | *Angelica pubescens* | Native | G | Anemochory |
|  | *Hydrocotyle ramiflora* | Native | G | Barochory |
|  | *Libanotis ugoensis* | Native | G | Barochory |
| *Apocynaceae* | |  |  |  |
|  | *Vincetoxicum pycnostelma* | Native | G | Anemochory |
| *Araliaceae* | |  |  |  |
|  | *Aralia cordata* | Native | G | Endozoochory |
| *Asparagaceae* | |  |  |  |
|  | *Convallaria majalis* | Native | G | Endozoochory |
|  | *Hosta sieboldiana* | Native | G | Anemochory |
|  | *Polygonatum odoratum* | Native | G | Endozoochory |
| *Asteraceae* | |  |  |  |
|  | *Achillea alpina* | Native | G | Barochory |
|  | *Ambrosia trifida* | Exotic | G | Epizoochory |
|  | *Anaphalis margaritacea* | Native | G | Anemochory |
|  | *Artemisia indica* | Native | G | Barochory |
|  | *Artemisia japonica* | Native | G | Barochory |
|  | *Aster glehnii* | Native | G | Anemochory |
|  | *Aster microcephalus* | Native | G | Anemochory |
|  | *Cirsium japonicum* | Native | G | Anemochory |
|  | *Cirsium makinoi* | Native | G | Anemochory |
|  | *Cirsium oligophyllum* | Native | G | Anemochory |
|  | *Erigeron annuus* | Exotic | G | Anemochory |
|  | *Erigeron strigosus* | Exotic | G | Anemochory |
|  | *Erigeron thunbergii* | Native | G | Anemochory |
|  | *Eupatorium glehnii* | Native | F | Anemochory |
|  | *Hieracium umbellatum* | Native | G | Anemochory |
|  | *Hypochaeris radicata* | Exotic | G | Anemochory |
|  | *Inula salicina* | Native | G | Anemochory |
|  | *Ixeridium dentatum* | Native | G | Anemochory |
|  | *Ligularia dentata* | Native | G | Anemochory |
|  | *Petasites japonicus* | Native | F | Anemochory |
|  | *Picris hieracioides* | Native | G | Anemochory |
|  | *Senecio cannabifolius* | Native | G | Anemochory |
|  | *Senecio nemorensis* | Native | F | Anemochory |
|  | *Serratula coronata* | Native | G | Anemochory |
|  | *Solidago virgaurea* | Native | G | Anemochory |
|  | *Synurus excelsus* | Native | G | Anemochory |
|  | *Taraxacum officinale* | Exotic | G | Anemochory |
| *Balsaminaceae* | |  |  |  |
|  | *Impatiens noli-tangere* | Native | G | Ballochory |
| *Betulaceae* | |  |  |  |
|  | *Betula platyphylla* | Native | F | Anemochory |
| *Brassicaceae* | |  |  |  |
|  | *Barbarea vulgaris* | Exotic | G | Barochory |
|  | *Cardamine leucantha* | Native | F | Ballochory |
| *Campanulaceae* | |  |  |  |
|  | *Adenophora triphylla* | Native | G | Barochory |
|  | *Campanula punctata* | Native | F | Barochory |
|  | *Platycodon grandiflorus* | Native | G | Barochory |
| *Cannabaceae* | |  |  |  |
|  | *Humulus scandens* | Native | F | Myrmecochory |
| *Caprifoliaceae* | |  |  |  |
|  | *Patrinia scabiosifolia* | Native | G | Barochory |
|  | *Patrinia villosa* | Native | G | Barochory |
|  | *Scabiosa japonica* | Native | G | Barochory |
| *Caryophyllaceae* | |  |  |  |
|  | *Arenaria lateriflora* | Native | G | Myrmecochory |
|  | *Cerastium rubescens* | Native | F | Barochory |
| *Celastraceae* | |  |  |  |
|  | *Celastrus orbiculatus* | Native | F | Endozoochory |
| *Chloranthaceae* | |  |  |  |
|  | *Chloranthus japonicus* | Native | F | Barochory |
| *Crassulaceae* | |  |  |  |
|  | *Phedimus aizoon var. floribundus* | Native | F | Barochory |
| *Cyperaceae* | |  |  |  |
|  | *Carex lanceolata* | Native | G | Barochory |
|  | *Carex leucochlora* | Native | G | Myrmecochory |
|  | *Carex nervata* | Native | G | Barochory |
| *Dennstaedtiaceae* | |  |  |  |
|  | *Pteridium aquilinum* | Native | G | Anemochory |
| *Dioscoreaceae* | |  |  |  |
|  | *Dioscorea nipponica* | Native | F | Anemochory |
| *Dryopteridaceae* | |  |  |  |
|  | *Dryopteris erythrosora* | Native | F | Anemochory |
| *Equisetaceae* | |  |  |  |
|  | *Equisetum arvense* | Native | G | Anemochory |
| *Fabaceae* | |  |  |  |
|  | *Amphicarpaea bracteata* | Native | G | Ballochory |
|  | *Lespedeza bicolor* | Native | G | Barochory |
|  | *Lespedeza cuneata* | Native | G | Barochory |
|  | *Trifolium dubium* | Exotic | G | Barochory |
|  | *Trifolium pratense* | Exotic | G | Barochory |
|  | *Trifolium repens* | Exotic | G | Barochory |
| *Gentianaceae* | |  |  |  |
|  | *Gentiana scabra* | Native | G | Barochory |
|  | *Gentiana zollingeri* | Native | G | Hydrochory |
|  | *Halenia corniculata* | Native | G | Barochory |
| *Geraniaceae* | |  |  |  |
|  | *Geranium thunbergii* | Native | G | Ballochory |
| *Hydrangeaceae* | |  |  |  |
|  | *Hydrangea paniculata* | Native | F | Anemochory |
| *Hypericaceae* | |  |  |  |
|  | *Hypericum ascyron* | Native | F | Barochory |
|  | *Hypericum erectum* | Native | G | Barochory |
| *Iridaceae* | |  |  |  |
|  | *Iris sanguinea* | Native | G | Barochory |
| *Juncaceae* | |  |  |  |
|  | *Luzula multiflora* | Native | G | Myrmecochory |
| *Lamiaceae* | |  |  |  |
|  | *Clinopodium chinense* | Native | G | Barochory |
|  | *Clinopodium gracile* | Native | F | Barochory |
|  | *Isodon umbrosus* | Native | F | Barochory |
|  | *Prunella vulgaris* | Native | G | Barochory |
|  | *Thymus quinquecostatus* | Native | G | Anemochory |
| *Liliaceae* | |  |  |  |
|  | *Lilium leichtlinii* | Native | G | Barochory |
| *Lycopodiaceae* | |  |  |  |
|  | *Lycopodium clavatum* | Native | G | Anemochory |
| *Onagraceae* | |  |  |  |
|  | *Chamerion angustifolium* | Native | G | Anemochory |
|  | *Oenothera biennis* | Exotic | G | Barochory |
| *Ophioglossaceae* | |  |  |  |
|  | *Botrychium ternatum* | Native | G | Anemochory |
|  | *Ophioglossum vulgatum* | Native | G | Anemochory |
| *Orchidaceae* | |  |  |  |
|  | *Liparis japonica* | Native | F | Anemochory |
| *Pinaceae* | |  |  |  |
|  | *Larix kaempferi* | Native | F | Anemochory |
| *Plantaginaceae* | |  |  |  |
|  | *Plantago asiatica* | Native | G | Epizoochory |
|  | *Plantago lanceolata* | Exotic | G | Epizoochory |
|  | *Veronica onoei* | Native | G | Anemochory |
|  | *Veronicastrum japonicum* | Native | G | Barochory |
| *Poaceae* | |  |  |  |
|  | *Anthoxanthum odoratum* | Exotic | G | Barochory |
|  | *Arthraxon hispidus* | Native | G | Barochory |
|  | *Arundinella hirta* | Native | G | Barochory |
|  | *Dactylis glomerata* | Exotic | G | Barochory |
|  | *Festuca ovina* | Native | G | Barochory |
|  | *Festuca rubra* | Exotic | G | Barochory |
|  | *Miscanthus sinensis* | Native | G | Anemochory |
|  | *Phleum pratense* | Exotic | G | Barochory |
|  | *Poa sphondylodes* | Native | G | Barochory |
|  | *Sasa senanensis* | Native | F | Barochory |
|  | *Schedonorus phoenix* | Exotic | G | Barochory |
|  | *Zoysia japonica* | Native | G | Barochory |
| *Polygalaceae* | |  |  |  |
|  | *Polygala japonica* | Native | G | Myrmecochory |
| *Polygonaceae* | |  |  |  |
|  | *Fallopia japonica* | Native | G | Anemochory |
|  | *Persicaria longiseta* | Native | G | Barochory |
|  | *Persicaria sagittata* | Native | G | Barochory |
|  | *Polygonum thunbergii* | Native | F | Barochory |
|  | *Rumex acetosella* | Exotic | G | Barochory |
|  | *Rumex japonicus* | Native | G | Anemochory |
| *Primulaceae* | |  |  |  |
|  | *Lysimachia clethroides* | Native | G | Barochory |
|  | *Lysimachia japonica* | Native | G | Barochory |
| *Ranunculaceae* | |  |  |  |
|  | *Aquilegia buergeriana* | Native | G | Barochory |
|  | *Ranunculus japonicus* | Native | G | Barochory |
|  | *Thalictrum aquilegiifolium* | Native | G | Barochory |
|  | *Thalictrum minus* | Native | G | Barochory |
| *Rosaceae* | |  |  |  |
|  | *Agrimonia pilosa* | Native | G | Epizoochory |
|  | *Potentilla cryptotaeniae* | Native | G | Barochory |
|  | *Potentilla fragarioides* | Native | G | Myrmecochory |
|  | *Potentilla freyniana* | Native | G | Myrmecochory |
|  | *Rosa amblyotis* | Native | G | Endozoochory |
|  | *Rosa multiflora* | Native | G | Endozoochory |
|  | *Rubus parvifolius* | Native | G | Endozoochory |
|  | *Rubus subcrataegifolius* | Native | G | Endozoochory |
| *Rubiaceae* | |  |  |  |
|  | *Galium japonicum* | Native | F | Epizoochory |
|  | *Galium trachyspermum* | Native | G | Barochory |
|  | *Galium verum* | Native | G | Barochory |
| *Salicaceae* | |  |  |  |
|  | *Populus tremula* | Native | F | Anemochory |
|  | *Salix chaenomeloides* | Native | F | Anemochory |
|  | *Salix integra* | Native | F | Anemochory |
| *Santalaceae* | |  |  |  |
|  | *Thesium chinense* | Native | G | Barochory |
| *Sapindaceae* | |  |  |  |
|  | *Acer crataegifolium* | Native | F | Anemochory |
| *Saxifragaceae* | |  |  |  |
|  | *Astilbe thunbergii* | Native | F | Barochory |
|  | *Rodgersia podophylla* | Native | F | Anemochory |
| *Schisandraceae* | |  |  |  |
|  | *Schisandra chinensis* | Native | F | Endozoochory |
| *Thelypteridaceae* | |  |  |  |
|  | *Thelypteris phegopteris* | Native | F | Anemochory |
| *Violaceae* | |  |  |  |
|  | *Viola grypoceras* | Native | G | Myrmecochory |
|  | *Viola mandshurica* | Native | G | Myrmecochory |
